# U-Shaped Relation between Plasma Oxytocin Levels and Behavior in the Trust Game

**DOI:** 10.1371/journal.pone.0051095

**Published:** 2012-12-05

**Authors:** Songfa Zhong, Mikhail Monakhov, Helen P. Mok, Terry Tong, Poh San Lai, Soo Hong Chew, Richard P. Ebstein

**Affiliations:** 1 Department of Economics, National University of Singapore, Singapore, Singapore; 2 Department of Obstetrics & Gynaecology, Yong Loo Lin School of Medicine, National University Health System, National University of Singapore, Singapore, Singapore; 3 Department of Paediatrics, National University of Singapore, Singapore, Singapore; 4 Department of Economics and Department of Finance, National University of Singapore, Singapore, Singapore; 5 Department of Psychology, National University of Singapore, Singapore, Singapore; University of Regensburg, Germany

## Abstract

Trust underpins much of social and economic exchanges across human societies. In experimental economics, the Trust Game has served as the workhorse for the study of trust in a controlled incentivized setting. Recent evidence using intranasal drug administration, aka ‘sniffing’, suggests that oxytocin (OT) can function as a social hormone facilitating trust and other affiliative behaviors. Here we hypothesized that baseline plasma OT is a biomarker that partially predicts the degree of trust and trustworthiness observed in the trust game. Using a large sample of 1,158 participants, we observed a significant U-shaped relationship between plasma OT with the level of trust, and marginally with the level of trustworthiness, especially among males. Specifically, subjects with more extreme levels of plasma OT were more likely to be trusting as well as trustworthy than those with moderate levels of plasma OT. Our results contribute to a deeper understanding of the biological basis of human trust and underscore the usefulness of peripheral plasma OT measures in characterizing human social behavior.

“*You must trust and believe in people or life becomes impossible*.” Anton Chekhov.

## Introduction

It is difficult to overstate the role of trust in facilitating the smooth functioning of social and market institutions in modern societies. Trust can be seen to provide the basis for reducing social complexity [1], enhancing social order [2] and social capital [3], as well as overcoming the inherent risk involved in economic exchange and social interaction [4]. In experimental economics, Berg, Dickhaut, and McCabe (1995) invented an economic game, called the Trust Game (TG) in which the first mover is endowed with certain amount of money, and can send any part of it to the second player, called the trustee, which is endowed with no money. The amount received by the trustee is typically tripled the amount sent. The trustee has the option to send any proportion of the tripled amount to the first mover, and keep the rest. Notice that the amount sent by the first mover can be a measure of the degree of trust while the amount sent by the trustee back to the first mover can be a measure of trustworthiness. The TG provides invaluable insights into many basic concepts in human relationships and demonstrates that “reciprocity exists as a basic element of human behavior which is accounted for in the trust extended to an anonymous counterpart” [5]. Since its inception, the incentivized TG has served as the mainstay for the study of trust in the controlled laboratory setting. More recently, the burgeoning field of neuroeconomics has begun to use this game to examine the biological underpinnings of trust [5].

Remarkably, using the TG in the laboratory has enabled the identification of the nonapeptide hormone, oxytocin (OT) as a promoter of trust related behavior. A series of experiments initiated by the seminal study of Kosfeld et al [6] showed that intranasal administration of OT enhances trust but not trustworthiness in the TG. Altogether, a growing body of work has now demonstrated the robust effect of intranasal administration of OT on trust related behaviors. Notably, the effects of sniffing OT on face recognition and in-group trust are significant in recent meta-analysis [7]. Similarly, a comprehensive literature review of the effects of sniffing OT showed that release of this peptide correlates with behavioral changes [8].

In the brain, the main source of OT is the magnocellular neurons of the paraventricular (PVN) and supraoptic (SON) nuclei of the hypothalamus. From these nuclei this hormone reaches the posterior pituitary by axonal transport and is released into the peripheral circulation where it regulates a number of critical physiological processes including parturition and lactation [9]. Importantly, OT is also released from neuronal dendrites and acts at distant brain targets [10]. In the last decade, accumulating evidence shows that this neuropeptide is important in shaping human social cognition and affiliative behaviors [11]. Towards revealing the role of OT in humans, intranasal administration, aka ‘sniffing’, has been a widely used strategy in understanding the action of this hormone in both normal [6], [12] and abnormal [13], [14] social behaviors. Neurogenetic strategies have also been very effectively used in unravelling the role of OT in autistic disorders as well as prosocial behavior in non-clinical subjects [15], [16], with some exceptions [17], [18].

Another widely used strategy in characterizing the role of OT in human social behaviors is the determination of OT levels in the peripheral circulation, albeit the contours of the relationship between plasma OT and CNS oxytocin remain unclear [19]. Indeed, plasma OT is also likely to partially reflect peripheral release of this neuropeptide, however, with no less relevance we suggest to the social brain [8]. Notably, plasma OT level has been shown to be remarkably stable over time. For example, OT levels at early pregnancy and the postpartum period are highly correlated at more than 90% [20]. Although there are technical issues relating to the measurement of plasma OT that remain to be resolved [21], many investigations have reported intriguing correlations between plasma OT and a wide range of behaviors including parent-infant bonding, adult pair-bonding and social relationships among others (reviewed by [8]).

It is the importance of trust and reciprocity in human social exchanges coupled with the considerable evidence that plasma OT levels are related, however non-linearly, to human social behavior, which prompts the current investigation. We are aware of only two previous studies that examined plasma OT levels in relation to trust and trustworthiness using the TG in a laboratory-based setting. Zak et al [22] used a sequential anonymous TG with monetary payoffs with 156 subjects. They find that OT levels rise in subjects who receive a monetary transfer that reflects an intention of trust relative to an unintentional monetary transfer of the same amount. Keri and Kiss [23] used a non-conventional trust paradigm mimicking everyday intimate transactions with 60 subjects and showed that OT was elevated in the trust-related condition relative to a neutral baseline. They also observed a significant positive relationship between trust-related oxytocin level and habituation of autonomic arousal.

Here, we hypothesized that base-line plasma OT, which is correlated with a range of human behaviors, is a biomarker for trust and trustworthiness, beliefs that underpin most human exchanges. To test this hypothesis we measured base-line plasma OT levels in a very large sample of 1,158 undergraduate Han Chinese students at the National University of Singapore who participated in one-shot TG. Notably, this investigation represents the largest sample of subjects yet examined for plasma OT levels in a single study.

**Figure 1:**
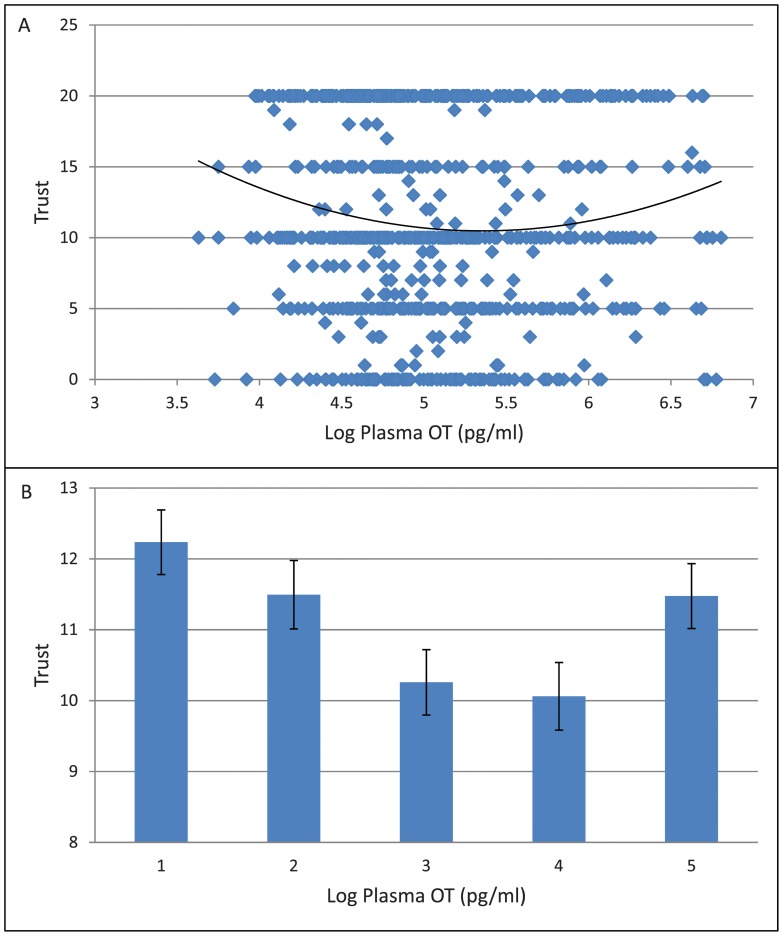
Plasma oxytocin and trust. (A) Scatter Plot on the relationship between plasma oxytocin and trust. (B) Histogram on the relationship between plasma oxytocin and trust.

### Ethics Statement

The study is approved by the Internal Review Board at the National University of Singapore. Each participant provides written informed consent (as outlined in the PLoS consent form) to participate in the experiment at the beginning of the study.

## Materials and Methods

### Subjects

We recruited 1,158 (584 females; age, mean 21.2± S.D. 1.5) Han Chinese undergraduate students at the National University of Singapore to participate in a study of the biological basis of human behavior through advertisement on the Integrated Virtual Learning Environment. At the beginning of the experiment, subjects completed an informed consent form approved by the Institutional Review Board at National University of Singapore. Subsequently, subjects participated in a 2-hour testing session to complete various tasks including the trust game and risk attitude without any feedback in a fixed order using paper and pencil. At the end of the experiment, two out of 20 tasks are randomly drawn to pay the subjects. Several days later, subjects donated 10 to 20 cc of blood for analysis including plasma oxytocin.

**Table 1:** Regression results for linear and nonlinear relationship between plasma oxytocin and trust.

		*model 1*			*model 2*		
		*pool*	*male*	*female*	*pool*	*male*	*female*
***oxytocin***	***Coeficient***	−0.319	−0.329	−0.268	−15.741	−25.215	−7.008
	***Std.Err***	0.348	0.575	0.437	4.921	8.074	5.851
	***P-value***	0.360	0.568	0.540	0.001	0.002	0.232
***oxytocin_ square***	***Coeficient***				1.465	2.385	0.637
	***Std.Err***				0.467	0.768	0.553
	***P-value***				0.002	0.002	0.250
***_cons***	***Coeficient***	12.724	13.176	12.094	52.703	77.154	29.674
	***Std.Err***	1.774	2.862	2.256	12.830	20.977	15.334
	***P-value***	0.001	0.001	0.001	0.001	0.001	0.053
***R-squared***		0.001	0.001	0.001	0.01	0.022	0.003
***Observations***		1061	508	536	1061	508	536

The table reports coefficient, standard error, and p values. The last row is R-squared.

### Behavioral Design

In the trust game, the first player is endowed with SGP$ 20 (about US$16), while the second player is endowed with nothing. In the first stage, the first player decides how much to send (*S*) to an anonymous and randomly matched second player (*20 – S*). For every dollar the first player sends, the second player receives three times (*3S*). In the second stage, the second player decides how much (*B*) out of *3S* to second back to the first player. At the end, the first player receives (*20 – S + B*) being the amount he/she keeps plus the amount the second player sends back while the second player receives three times the amount sent deducting the amount sent back (*3S – B*). We use the strategic method [24], in which the second player states his/her response to each of 21 possible choices from the first player. Every participant plays both roles of first and second players without any feedback. At the payment stage with real money, we randomly determine the specific role – first or second mover – for each pair of subjects. The amount sent by first player is used as a measure for trust while the average return amount from the second player is a measure for trustworthiness.

As trust is an inherently risky behavior viz., the trustee may not reciprocate with an act of trustworthiness, we aim to test whether OT is specific to trust in the social interactions or alternatively plasma OT indexes risk in general. We therefore include a risk task using portfolio choice design [25]. In this risk task, subjects are endowed with SGD $20, and decide how much to invest on an experimental stock. For the amount invested, there is 50% chance that it will become 2.5 times, and 50% chance that it will become zero. This design enables us to observe different levels of risk aversion for each subject, and allows ascertainment of the specificity of plasma OT levels towards trust without including a confound i.e. a possible connection between OT and risk attitude.

### Assay Procedures

Blood samples for oxytocin assay were collected from the antecubital vein into pre-chilled 5 ml EDTA tubes with 250 KIU of apoprotinin, and refrigerated until processing. Plasma was isolated by centrifugation at 1800 g, 15 minutes, 4°C, and stored in aliquots at −70°C. Oxytocin immunoreactivity levels were quantified in duplicates using a commercial oxytocin ELISA kit (Enzo Life Sciences, NY, USA, formerly Assays Designs, MI, USA), as recommended in previous publications [11]. Thawed samples on ice were diluted 1∶2 times in assay buffer and assayed according to manufacturer's instructions. The oxytocin assay had a sensitivity of 11.7 pg/ml, and inter- and intra-assay coefficient of variations below 15%.

Currently there are differences in opinions surrounding the measurement of oxytocin and particularly concerning the requirement of sample extraction. The commercially available oxytocin EIA kit from Enzo Life (formerly Assay Designs), which has been validated by for linearity, cross reactivity, matrix effects, accuracy, precision and recovery [26], was used in the current study. The experience of some investigators suggests that extraction of the samples leads to significant loss of measureable oxytocin. Importantly, the oxytocin data from non-extracted samples makes biological sense as compared to those from extracted samples, which often gave rise to non-detectable levels of oxytocin. Szeto et al., 2011 confirmed these technical findings; as much as two-three fold of the authentic oxytocin was removed by extraction, and 5% of the extracted samples had non-detectable oxytocin levels [21]. Moreover, it would not be appropriate to measure extremely low levels of target analyte, viz., following extraction in the case of oxytocin, using commercial immunoassays that are insufficiently sensitive, which gives rise to erroneous results. We are also not keen to extract the samples using the solid-phase extraction method, as the procedure requires large volume of samples, and often gives rise to low recovery of analytes, high variability in results and incomplete removal of interferences. Hence, we chose not to extract. During our assay runs, we performed 1∶2 dilutions on the unextracted samples so that the measured oxytocin concentrations fall within the “measurable” portion of the standard curve. Pre-dilution of samples is also a common technical approach used to reduce assay interference due to sample matrix.

Taking into consideration the labile nature of oxytocin in biological matrix [27], we followed strict protocol during sample collection to limit its enzymatic breakdown. All blood samples were collected into pre-chilled EDTA tubes containing protease inhibitor. Processing of samples was performed at 4 deg C. During assay, thawed samples were kept on ice. In Szeto et al., human EDTA plasma/serum were obtained and stored at −80 deg C until assay. There was no mention of proper sample collection and hence, the stability of oxytocin in their plasma samples needs to be examined, which may offer one possible explanation of the degraded oxytocin products found in their samples.

The concentrations of oxytocin in unextracted blood samples are 100-fold more than in extracted samples [21]. Martin-Protean, a biotechnology company that specialises in protein analysis, reported oxytocin values to be higher (at levels of 1000 pg/ml) using novel isolation methods and mass spectrometry, and proposed a new model of explanation that incorporates oxytocin carrier protein neurophysin 1 [28]. In their website (http://martin-protean.com/), “Efforts to quantify oxytocin that capture 0.1% of the oxytocin present would not capture the complete oxytocin story and are likely to be dominated by non-biological variation in experimental procedure”.

Szeto et al. did perform stability tests using tritiated oxytocin added to plasma, and found that oxytocin is stable under different temperatures and after multiple freeze/thaw cycles. On the other hand, they concluded that plasma oxytocin has a short half life of 3–6 minutes and rapidly degrade into products that are more stable underscoring the careful handling procedure employed in our study. While Szeto et al. reported a lack of correlation between oxytocin levels in extracted versus non-extracted plasma samples (r = 0.09), another study reported high correlation (r = 0.89) [29].

Szeto et al. concluded that degradation products of oxytocin are likely to contribute to the measured levels of oxytocin in unextracted samples. However, it is doubtful that these are degraded products of oxytocin as they have molecular masses more than that of oxytocin. Based on their chromatography results, we believe there is a strong possibility that the assay is measuring immunoreactive oxytocin that comprises authentic oxytocin as well as oxytocin prohormones (OX-T) [30] or other forms of oxytocin in the unextracted samples. Recently, a novel form of oxytocin has been described in multiple species of squirrel monkeys, with a substitution of a leucine to a proline in amino acid position 8 [31]. The variant forms of oxytocin cannot be disregarded and appear to be biologically significant, as many other researchers have used the same kit to measure oxytocin in unextracted samples and found a myriad of associations with relevant physiological outcomes.

### Statistics

To examine the relationship between plasma OT, Trust and Trustworthiness, we used regression models with robust standard error in STATA 11. In the first model, only plasma OT is included in the analysis to test a linear effect of OT on trust behaviors. Since U-shaped dose response curves are often observed for the action of peptide neuromodulators and steroid hormone actions in the brain [32], [33], we added a quadratic in a second model to test for nonlinear relationships between plasma OT and trust related behaviors. In addition, as pointed out by a recent paper [34], the data in Zak et al [22] might indeed suggest a revered U-shaped relationship between plasma oxytocin and trust. Here, a significantly positive quadratic term would indicate a U-shaped relationship while a significant negative quadratic term would indicate an inverse U-shaped relationship. To examine gender effects, we carried out a separate analysis for each gender. All the statistics reported here are two-tailed.

## Results

### Behavioral parameters

The average amount sent from the first player (Trust) is 11.1 out of 20, while the average amount sent back to the second player (Trustworthiness) is 10.0 (Figure S1A, Figure S1B). This is similar to what is generally observed in the TG literature: the first player sends about 50% of his/her endowment and the second player sends back about 95% of what was sent (see [24] for a survey). In a linear regression, males significantly trust more than females (p<0.039), while we observe no significant gender difference in trustworthiness (p>0.363) (Figure S2A and Figure S2B). Our results are distinct from [35] which reported significant gender difference in trustworthiness but not in trust. This discrepancy likely indicates slight cultural differences across populations.

### Plasma OT levels

The average oxytocin level is 214± SD 230 pg/ml (Figure S1C) which is representative of the assay procedure used in the current investigation [8]. We test whether age and gender might have an effect on plasma OT level. As usually observed [36], [37], in linear regression, there is no significant gender difference in plasma OT levels (p>0.365) (Figure S2C), and also no significant age effect on Plasma OT (p>0.650). Following other investigations [38], [39], we exclude 23 subjects whose plasma OT exceed 3 standard deviations (>904) from the subsequent analysis (Figure S1C), and then use the log transformation in the analysis.

### Relationship between plasma OT, Trust and Trustworthiness

We test the effect of plasma OT on the levels of trust and trustworthiness in the TG using both linear and non-linear regression analysis; the results are summarized in Tables 1 and 2. Neither Trust nor Trustworthiness shows a significant linear relationship with plasma OT (Table 1, Table 2) whereas a significant non-linear U-shaped (quadratic) relationship is observed with Trust (OT, p<0.001; OT quadratic, p<0.002) (Figure 1A, 1B). Subjects in the top 20% and the bottom 20% of the plasma OT distribution “trust” on the average 15.6% more than those subjects in the middle 20% of the distribution (Figure 1B). Hence, subjects characterized at the extremes of plasma OT concentrations are significantly more trusting. After separating the analysis by sex, the significant relationship is observed only in male subjects (oxytocin: p<0.002; oxytocin quadratic: p<0.002), but not in female subjects (OT: p<0.232; OT quadratic: p<0.250) albeit in the joint analyses greater significance is observed.

**Table 2:** Regression results for linear and nonlinear relationship between plasma oxytocin and trustworthiness.

		*model 1*			*model 2*		
		*pool*	*male*	*female*	*pool*	*male*	*female*
***oxytocin***	***Coeficient***	−0.127	−0.036	−0.361	−7.951	−12.486	−5.514
	***Std.Err***	0.272	0.463	0.322	3.885	6.759	4.396
	***P-value***	0.641	0.939	0.278	0.041	0.065	0.210
***oxytocin_ square***	***Coeficient***				0.743	1.194	0.487
	***Std.Err***				0.367	0.646	0.413
	***P-value***				0.043	0.065	0.240
***_cons***	***Coeficient***	10.597	9.938	11.952	30.885	41.932	25.446
	***Std.Err***	1.380	2.311	1.651	10.159	17.499	11.541
	***P-value***	0.001	0.001	0.001	0.002	0.017	0.028
***R-squared***		0	0	0.002	0.005	0.009	0.005
***Observations***		990	481	491	990	481	491

The table reports coefficient, standard error, and p values. The last row is R-squared.

**Figure 2:**
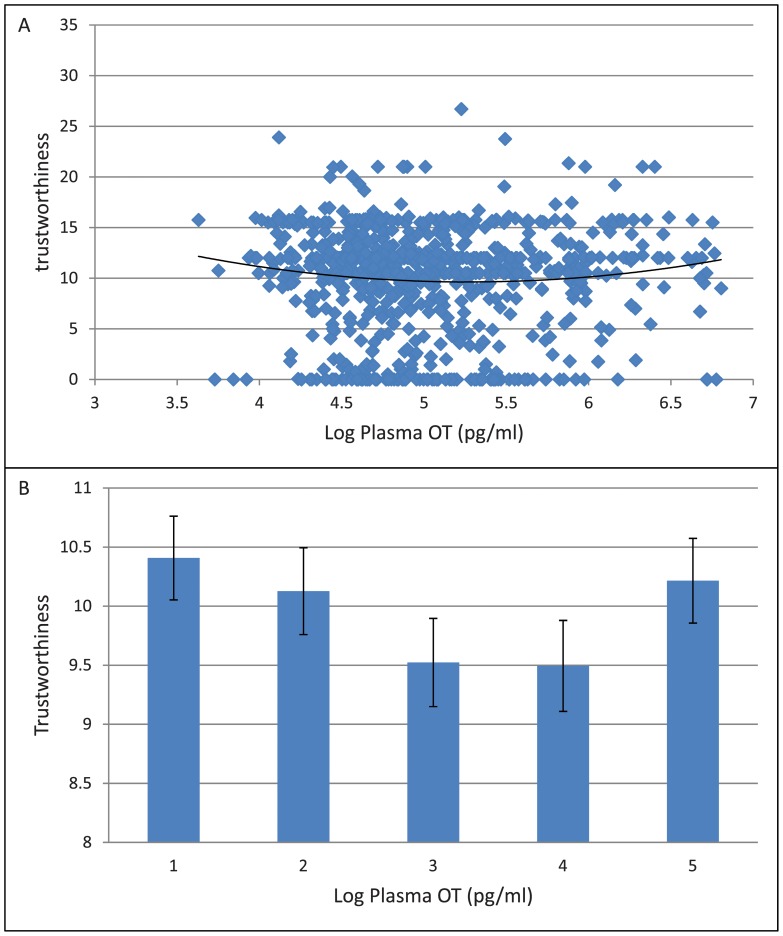
Plasma oxytocin and trustworthiness. (A) Scatter Plot on the relationship between plasma oxytocin and trustworthiness. (B) Histogram on the relationship between plasma oxytocin and trustworthiness.

For average Trustworthiness, we find a significant U-shaped relationship between plasma OT and Trust (OT, p<0.041; OT quadratic, p<0.043) (Figure 2A, 2B) similar to that observed for Trust. Subjects in the top 20% and bottom 20% of the plasma plasma OT distribution are 8.3% more trustworthy than those in the middle 20% plasma OT distribution (Figure 2B). In the analysis separating by sex, similar to what we observed for Trust, a marginally significant relationship is again observed only in the male subjects (OT: p<0.065; OT quadratic: p<0.065), but not in female subjects (OT: p<0.210; OT quadratic: p<0.240).

We check the robustness of the results after including those subjects with plasma OT higher than 3 times of standard deviations. Similarly, a significant non-linear U-shaped relationship is observed with Trust (OT, p<0.024; OT quadratic, p<0.028), and a marginally significant non-linear U-shaped relationship is observed with Trustworthiness (OT, p<0.071; OT quadratic, p<0.070). We further check the robustness of the results after controlling gender and age in the regression analysis. Similarly, a significant non-linear U-shaped relationship is observed with Trust (OT, p<0.002; OT quadratic, p<0.002), and a significant non-linear U-shaped relationship is observed with Trustworthiness (OT, p<0.026; OT quadratic, p<0.029).

We also consider whether there might be a relationship between the decision to send and return. In our data, 11.5% of the subjects send nothing, and 10.2% of the subjects return nothing. However, in the probit regression, we do not observe a significant relationship between plasma oxytocin and the decision to send (OT, p>0.839; OT quadratic, p>0.832), nor the decision to return (OT, p>0.892; OT quadratic, p>0.895). This might be due to lack of power as the data is skewed for the binary decisions. Alternatively, it might suggest that the plasma OT is more reflecting the degree of trust and trustworthiness, rather than per se trust or not, and trustworthy or not.

Trust by the first mover is risky since the trustee may not reciprocate the amount transferred. As anticipated, risk attitude and trust are significantly correlated (corr  = 0.141, p<0.001) and the direction is positive viz., subjects with a propensity to financial risk are more trusting in the TG. Towards testing the specificity of plasma OT levels as a measure of the level of trust rather than degree of risk tolerance, we conduct a regression analysis with risk attitude as an additional independent variable. The inverted U-shaped association between trust and plasma OT remains robust (the whole sample: OT, p<0.001; OT quadratic, p<0.001; male only: OT, p<0.002; OT quadratic, p<0.002; female: OT, p<0.121; OT quadratic, p<0.133). We further test the relationship between risk and OT; no significant relationship is found (the whole sample: OT, p<0.693; OT quadratic, p<0.594; male only: OT, p<0.695; OT quadratic, p<0.812; female: OT, p<0.247; OT quadratic, p<0.264). This further supports the view that the effect of OT is specifically on trust but not risk attitude.

## Discussion

We have examined whether baseline plasma OT is a biomarker which partially predicts the levels of trust and trustworthiness observed in the TG in a large Han Chinese undergraduate sample. Notably, we observed a U-shape relation between the levels of trust and marginally trustworthiness and subjects' baseline plasma OT level. Specifically, subjects with more extreme levels of plasma OT were more likely to be trusting as well as trustworthy than those with moderate levels of plasma OT. U-shape relationship in hormone and neurotransmitter action is a frequent observation and documented in [32], [40]. In the brain the U-shaped dose-response relationship is particularly characteristic of peptide hormones often presented as an inverted U [41], [42] and sometimes as a simple U shape [32], [33] as observed in the current investigation.

We suggest the notion that the current findings, viz., the *extremes* of plasma OT predict trust and trustworthiness, reflect the complexities of the mechanisms underlying the role of OT in social cognition and especially, but not exclusively, the trust phenotype. As Bartz et al underscore in a cogent and recent review [43], the effects of OT are constrained by features of situations – simply put ‘context matters’. Indeed, the role of OT on social cognition and prosociality like trust/trustworthiness is nuanced and subtle. The current findings are consistent with this view and, moreover, help refine predictions about plasma OT levels and how this measure can inform the understanding of social cognition. Although a superficial view of OT actions might at first suggest a situation-invariant effect of this hormone on behavior, closer scrutiny suggests that the effects of OT are often moderated by contextual factors, and perhaps equally importantly, by trait characteristics of the subjects themselves. This scenario is not unique to OT. A good example is provided by the paradoxical effect of the stimulant methylphenidate in children with attention deficit; in these hyperactive children an amphetamine (“speed”) like drug has a calming effect [44]. Similarly, paradoxical effects have been observed for positive modulators of the GABA-A receptor (benzodiazepines, barbiturates, alcohol, GABA steroids) which generally induce inhibitory (e.g. anesthetic, sedative, anticonvulsant, anxiolytic) effects but some individuals have adverse effects (seizures, increased pain, anxiety, irritability, aggression) upon exposure [45].

Evidence specifically supports such a non-linear role of OT tone on the complex trust phenotype. For example, a recent investigation shows that administered OT enhances cooperation and reduces betrayal aversion contingent on other personality factors [46]. OT has a non-linear effect on trust, cooperation and betrayal aversion contingent upon an individual's background personality trait of Attachment Avoidance. Similarly, such non-linear effects of OT on trust also characterize borderline personality disorder (BPD) [47]. Results showed that intranasal OT produced opposite actions in BPD (compared to the trust-enhancing effect of OT in normal subject), decreasing trust and the likelihood of cooperative responses. Moreover, U-shaped relationships between OT and behavior are not restricted to humans but have also been observed in animal studies. An especially relevant example has been reported for the role of OT in memory storage and consolidation in mice [48] and rats [49].

Summing up, the U shaped relationship herein observed between plasma OT and trust/trustworthiness is another example, we suggest, of how hormones overall, and OT specifically, may have paradoxically opposite actions contingent on individual differences. We suggest that the quadratic relationship between plasma OT and trust/trustworthiness captures the concept put forward by Bartz et al that ‘context and person matters’ in the action of this nonapeptide hormone [43]. In some individuals, low central OT tone reflected in low plasma OT levels, is associated with trust whereas in other individuals high plasma OT, presumably reflecting high central OT tone, is associated with trust. Bartz et al have suggested in their recent review that endogenous OT reflected in plasma measurements could be a biomarker of sensitivity to social cues and/or social motivation. Low plasma OT, which has been reported in autism [50], would reflect social insensitivity and motivation whereas high plasma OT could reflect increased social sensitivity and motivation. Hence, both low and high social sensitivity may drive trust/trustworthiness as observed in the current report. Low social sensitivity may make such individuals less betrayal averse and less fearful of exploitation and hence more likely to trust in the TG. Similarly, subjects with high plasma OT indicating increased social sensitivity and motivation would also show enhanced trust/trustworthiness driven by the warm glow of positive social interactions incurred in participating in the TG.

There is a large body of literature in evolutionary biology on gender differences regarding reproduction and investment cost towards offspring [51]. As females usually incur higher costs for reproduction including giving birth and raising offspring, it is suggeseted that females will increase their fitness (number of viable offspring) by investing heavily in fewer children but insuring their the survival. On the other hand, males would increase their fitness by accessing to the maximum number of females they can mate with since the male investment in child bearing and rearing is less than the female. The behavioral consequences of gender difference in reproduction are unclear. It is, however, possible that these different investment strategies could lead to different psychological mechanisms towards trust and trustworthiness but also different biological mechanisms underpinning these two constructs for each of the sexes. In our findings, although the relationship between plasma OT and trust related behavior is significant in the pooled sample, separate analysis by gender revealed that the effects are more specific to the male side. This is in agreement with a large number of animal studies suggesting that OT differentially affects females and males, likely reflecting the interactions between OT and gonadal hormones [52], [53]. Indeed, the effects of neuropeptides across different species show sexual dimorphic effects on behavior. For example, the parenting behavior of female prairie voles is more dependent on OT, whereas male parenting behavior is more associated with AVP [54]. Furthermore, there are gender differences in OT receptor binding depending on the brain area and the species. For instance, female prairie voles show higher binding of OT receptors in the medial prefrontal cortex compared to males [55]. Higher OT receptor binding was found in the hypothalamus of female rats compared to male rats [56]. Altogether then, it is not surprising that in the current investigation we observe a gender-specific relationship between plasma OT levels and the level of trust and marginally trustworthiness observed in the trust game.

In “All's Well That Ends Well” Shakespeare advised “Love all, trust a few, do wrong to none”. This sage advice recognizes the inherent risk of betrayal embodied in bestowing trust [57], [58], [59]. The TG is designed to measure trust but its measurement is confounded by the element of risk since we must also consider that trust is not reciprocated [60]. To minimize the confounding aspect of risk inherent in the TG, we also assessed subjects' risk attitude using a portfolio choice design [25]. The use of this design allowed us to decompose trust and achieve a more refined estimate of an individual's trusting behavior by minimizing potential confound of risk proneness or risk aversion. Our experimental design shows that plasma OT is reflecting trust and not risk attitude, as there appears to be no significant relationship between this hormone measure and risk. However, we are aware of the potential confound of trust and trustworthiness with other prosocial behaviors including altruism and inequity aversion [61]. As a consequence, our study needs to be interpreted with caution since the results we report might not necessarily reflect only trust and trustworthiness, but other facets of prosocial behaviors that are related to the role of plasma OT.

There are two caveats in place regarding measurement of plasma OT used in our study. The first question has to do with the laboratory method, for which we argue that our procedure is indeed reliable and robust for measuring plasma OT as detailed in the Methods section. Notwithstanding, we would also like to point out to the reader the report by Szeto et al [21], which raises the possibility that the procedure adopted by us and studies of others could be subjective to criticism and could be a potential limitation of the current investigation. However, the strengths of this study need also to be underscored viz., the careful measurement of the phenotype as well as the very large number of subjects examined. Secondly, whether plasma OT indeed is an informative measurement for CNS oxytocin remains unclear and needs to be fully resolved [19]. Many questions remain regarding how robustly and by what pathways (peripheral and central release) this biological marker reflects human social behavior. In the current report we interpret base-line plasma OT as a partial indicator or biomarker for neuropeptide ‘tone’ that reflects long-term chronic oxytocin activity. An alternative hypothesis proposed by Porges is that peripheral OT levels partially indexed in plasma levels could also be a measure in part of the vagal regulated ‘social engagement system’ [62]. Porges has suggested in an extensive series of publications that “the mammalian autonomic nervous system provides the neurophysiological substrates for the emotional experiences and affective processes that are major components of social behavior”. The role of OT in parasympathetic modulation, especially as a break on sympathetic heart activation, may facilitate prosocial behavior by establishing a calmer, less-threatening environment. Indeed, vagal tone predicts positive emotions and social connectedness [63]. Altogether, regardless of the source of plasma OT, peripheral or central release, there is good reason to believe that plasma OT levels is related albeit indirectly to social brain/social engagement. Nevertheless, there remain methodological issues surrounding the measurement of oxytocin and hence until these questions are resolved results using plasma measurements of this hormone, need to be interpreted cautiously.

Trust pervades human society and is a critical element in facilitating social interaction and exchange between individuals, groups, businesses, governments and nation states. It is therefore not unexpected that trust is the subject of intense inquiry by scholars across academic disciplines. Over the past decade, by examining trust through the lens of experimental economics, it has been possible to begin to unveil its neurobiological and neuroendocrinological underpinnings. Of special interest is the identification of OT, underpinned by a rich tradition of translational research in animal models [9], with trust in humans. The current report strengthens the link between OT and trust and most importantly, indicates that basal plasma levels of OT may serve as a provisional biomarker for trust and trustworthiness. Zak and Knack [64] have characterised the social, economic and institutional environments in which trust will be high, and show that low trust environments reduce the rate of investment. By exploring the neurobiological roots of trust and specifically showing that plasma OT may be a potential biomarker for human trust, we suggest the notion that a new window of investigation has opened regarding individual, cross cultural and cross group differences in trust related behavior.

## Supporting Information

Figure S1 **Distribution of trust behaviors and plasma oxytocin.** (A) Distribution of the amount sent by the first player; (B) Distribution of the average amount returned by the second player; (C) Distribution of level of oxytocin; (D) Distribution of log level of oxytocin without outliers.
(PDF)

Figure S2 **Gender Difference.** (A) Level of trust of male and female; (B) Level of trustworthiness of male and female; (C) Level of OT of male and female.
(PDF)
