# Supplementary material for: U-Shaped Relation between Plasma Oxytocin Levels and Behavior in the Trust Game
Source: PLoS One. 2012 Dec 5;7(12):e51095. doi: 10.1371/journal.pone.0051095 (PMC3515439; doi:10.1371/journal.pone.0051095)
Supplement: Figure S1 — Distribution of trust behaviors and plasma oxytocin. (A) Distribution of the amount sent by the first player; (B) Distribution of the average amount returned by the second player; (C) Distribution of level of oxytocin; (D) Distribution of log level of oxytocin without outliers. (PDF) [file pone.0051095.s001.pdf]

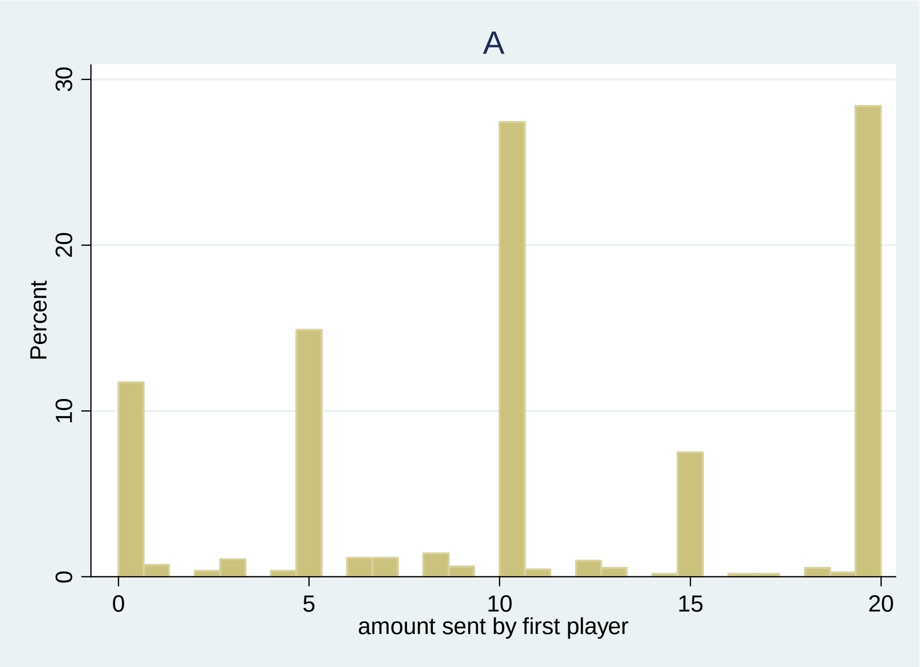
A
Percent
0
10
20
30
0
5
10
15
20
amount sent by first player

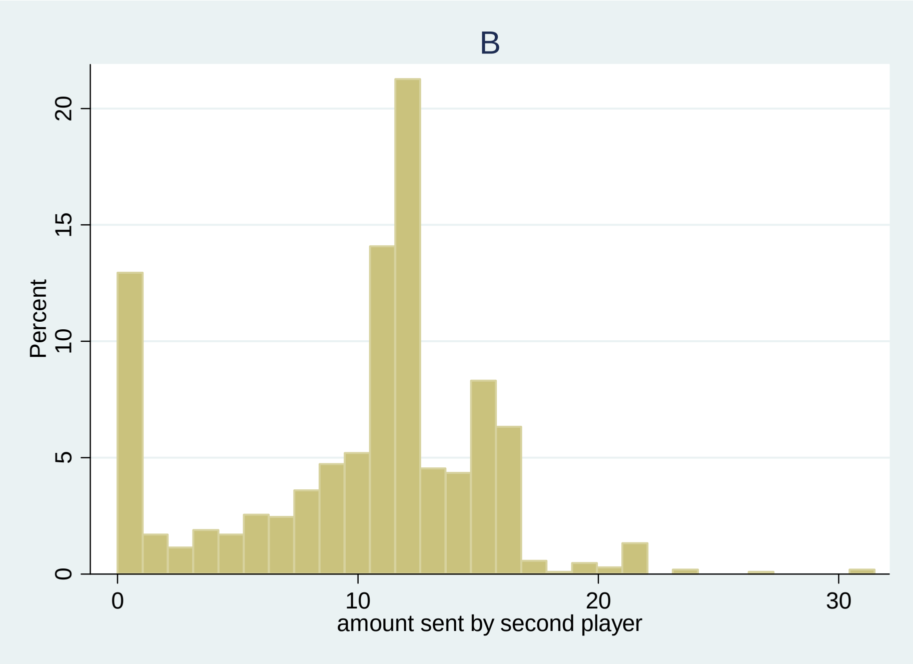
B
Percent
0
5
10
15
20
0
10
20
30
amount sent by second player

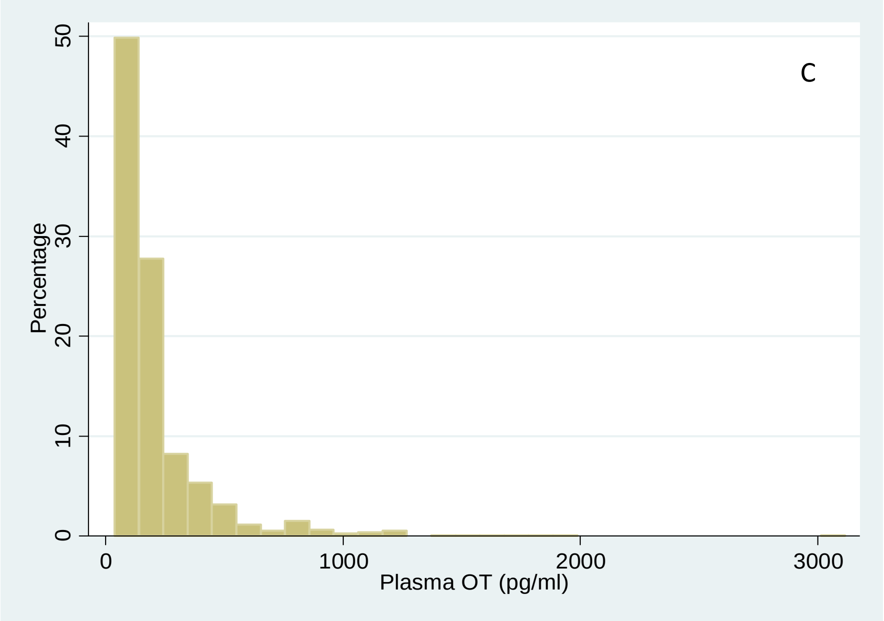

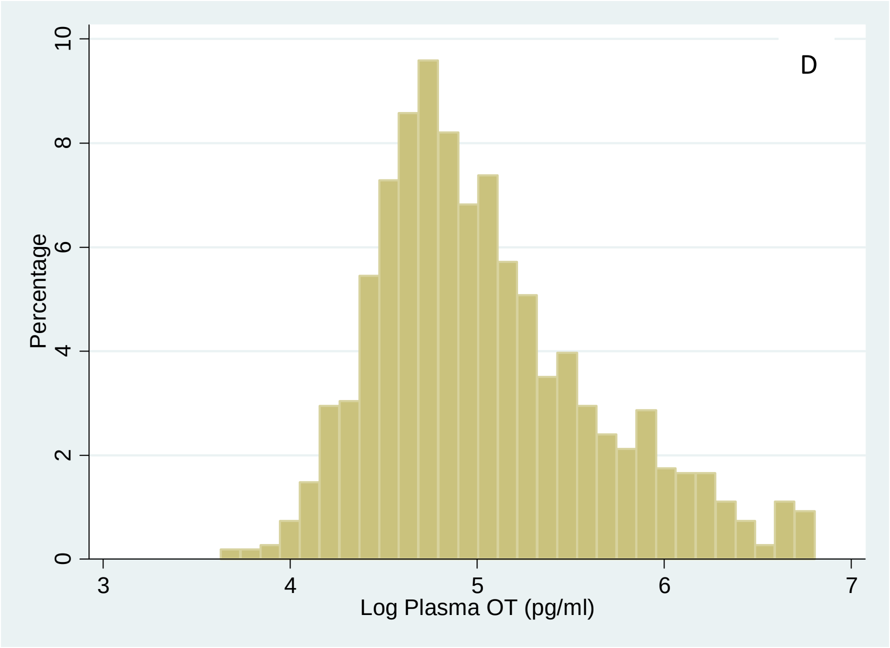

Figure S1. Distribution of trust behaviors and plasma oxytocin. (A) Distribution of the amount sent by the first player; (B) Distribution of the average amount returned by the second player; (C) Distribution of level of oxytocin; (D) Distribution of log level of oxytocin without outliers.
