# Supplementary material for: U-Shaped Relation between Plasma Oxytocin Levels and Behavior in the Trust Game
Source: PLoS One. 2012 Dec 5;7(12):e51095. doi: 10.1371/journal.pone.0051095 (PMC3515439; doi:10.1371/journal.pone.0051095)
Supplement: Figure S2 — Gender Difference. (A) Level of trust of male and female; (B) Level of trustworthiness of male and female; (C) Level of OT of male and female. (PDF) [file pone.0051095.s002.pdf]

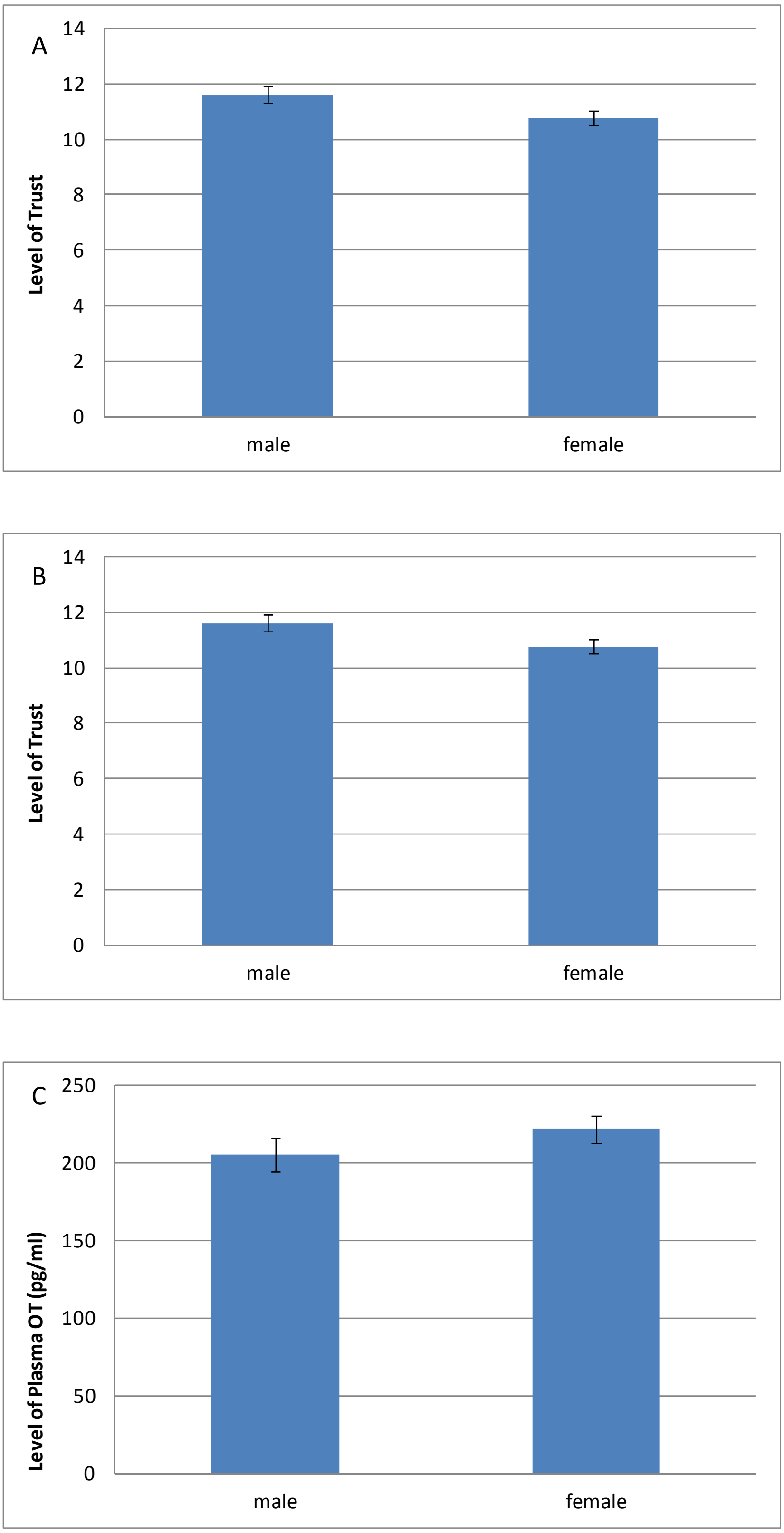

Figure S2. Gender Difference. (A) Level of trust of male and female; (B) Level of trustworthiness of male and female; (C) Level of OT of male and female.
